# Sulindac sulfide inhibits colon cancer cell growth and downregulates specificity protein transcription factors

**DOI:** 10.1186/s12885-015-1956-8

**Published:** 2015-12-16

**Authors:** Xi Li, Satya S. Pathi, Stephen Safe

**Affiliations:** Department of Veterinary Physiology & Pharmacology, Texas A&M University, College Station, TX 77843-4466 USA; Oklahoma Medical Research Foundation, 825 NE 13th St., Oklahoma City, OK 73104 USA

**Keywords:** Sulindac sulfide, Sp transcription factors

## Abstract

**Background:**

Specificity protein (Sp) transcription factors play pivotal roles in maintaining the phenotypes of many cancers. We hypothesized that the antineoplastic effects of sulindac and its metabolites were due, in part, to targeting downregulation of Sp transcription factors.

**Methods:**

The functional effects of sulindac, sulindac sulfone and sulindac sulfide on colon cancer cell proliferation were determined by cell counting. Effects of these compounds on expression of Sp1, Sp3, Sp4 and pro-oncogenic Sp-regulated genes were determined by western blot analysis of whole cell lysates and in transient transfection assays using GC-rich constructs.

**Results:**

Sulindac and its metabolites inhibited RKO and SW480 colon cancer cell growth and the order of growth inhibitory potency was sulindac sulfide > > sulindac sulfone > sulindac. Treatment of SW480 and RKO cells with sulindac sulfide downregulated expression of Sp1, Sp3 and Sp4 proteins. Sulindac sulfide also decreased expression of several Sp-regulated genes that are critical for cancer cell survival, proliferation and angiogenesis and these include survivin, bcl-2, epidermal growth factor receptor (EGFR), cyclin D1, p65 subunit of NFκB and vascular endothelial growth factor (VEGF). Sulindac sulfide also induced reactive oxygen species (ROS) and decreased the level of microRNA-27a in colon cancer cells, which resulted in the upregulation of the Sp-repressor ZBTB10 and this resulted in downregulation of Sp proteins.

**Conclusions:**

The results suggest that the cancer chemotherapeutic effects of sulindac in colon cancer cells are due, in part, to its metabolite sulindac sulfide which downregulates Sp transcription factors and Sp-regulated pro-oncogenic gene products.

## Background

Nonsteroidal anti-inflammatory drugs (NSAIDs) and cyclooxygenase (COX) inhibitors are widely used as analgesics and treatment of diseases associated with an inflammatory response, such as arthritis and cardiovascular diseases. Cancer has been associated with inflammation and there is epidemiologic evidence that NSAIDs decrease the risk for development of several cancers [1–3]. Several studies show that the use of aspirin and other NSAIDs is associated with decreased incidence of colon cancer, and aspirin use and treatment is also associated with a decrease in colon polyp formation [4–6]. Aspirin and NSAIDs such as sulindac decrease colon polyp formation and the latter compound has been used in several clinical studies for inhibition of polyp formation in colon cancer patients and genetically susceptible individuals [7–9].

Sulindac, a COX-1 and COX-2 inhibitor, has been extensively investigated as a potent chemotherapeutic drug for treatment of colon and other cancers; however, due to the metabolism of sulindac (sulfoxide) to its sulfone (oxidation) or sulfide (reduction) metabolites, the mechanisms of action and identity of the active compound(s) are uncertain. Several reports show that sulindac and its metabolites exhibit pronounced pro-apoptotic activity in cancer cell lines and animal models and this includes activation of both extrinsic and intrinsic apoptosis pathways [10–17]. For example, sulindac induced apoptosis in HT-29 colon cancer cells and this is related to downregulation of survivin which in turn is due to decreased expression of β-catenin which regulates survivin expression through the transcription factor TCF-4 [13]. Other studies also show downregulation of β-catenin and/or survivin in cancer cells and tumors treated with sulindac or its metabolites [15–17] and the pro-apoptotic effect of survivin downregulation in head and neck sarcoma and carcinoma cells is STAT2-dependent [11, 12].

In addition, it has also been reported that sulindac and its metabolites exhibit growth inhibitory activity and this is associated, in part, not only with downregulation of survivin but also decreased expression of the epidermal growth factor receptor (EGFR) and vascular endothelial growth factor (VEGF) [18–21]. Studies in this laboratory have demonstrated that basal expression of genes, such as survivin, VEGF and EGFR, in various cancer cell lines is dependent on specificity protein (Sp) transcription factors Sp1, Sp3 and Sp4 [22–26] which are highly expressed in many cancer cells and tumors [27]. In this study, we initially compared the growth inhibitory effects of sulindac and its metabolites in SW480 and RKO colon cancer cells and their order of activity was sulindac sulfide > sulindac sulfone > sulindac after treatment for 24, 48 or 72 h. At concentrations of sulindac and its metabolites that inhibited cell growth, we observed that only sulindac sulfide decreased levels of Sp1, Sp3 and Sp4 proteins and this was accompanied by decreased expression of Sp1-dependent genes such as VEGF, survivin, EGFR and bcl-2. Mechanistic studies suggest that sulindac sulfide induces reactive oxygen species (ROS) which in turn downregulates expression of Sp1, Sp3 and Sp4 in colon cancer cells [27].

## Methods

### Cell lines and cell culture, chemicals, oligonucleotides, plasmids and antibodies

SW480 and RKO colon cancer cell lines were obtained from the American Type Culture Collection (ATCC, Manassas, VA) and maintained in Dulbecco’s modified Eagle’s medium (DMEM) nutrient mixture with Ham’s F-12 (DMEM/Ham’s F-12) (Sigma-Aldrich, St. Louis, MO) as described [25]. Sulindac, sulindac sulfone and sulindac sulfide were purchased from LKT Laboratories (St. Paul, MN). Real-time PCR primer sequences for ZBTB10 are forward: 5'-GCT GGA TAG TAG TTA TGT TGC and reverse: 5'-CTG AGT GGT TTG ATG GAC AGA.

Sp1 construct (pSp1-Luc) contains the −751 to −20 bp promoter insert linked to a luciferase reporter gene [28] and Sp3 construct (pSp3-Luc) contains the −417 to −38 bp promoter insert [29]. Survivin construct (pSurvivin-Luc) contains the −220 to +49 bp promoter insert [30] and VEGF construct (pVEGF-Luc) contains the −2018 to +50 bp promoter insert [31] and miR-27a construct (pmiR-27a-Luc) contains the −603 to +36 bp promoter insert [32]. The empty luciferase vector pGL2 was also used in parallel as a negative control in luciferase assay. Sp1, survivin, cleaved PARP and β-actin antibodies were purchased from Upstate/Millipore (Lake Placid, NY), R&D Systems (Minneapolis, MN), Cell Signaling Technology (Beverly, MA) and Sigma-Aldrich, respectively. Sp3, Sp4, EGFR, p65 and VEGF antibodies were purchased from Santa Cruz Biotechnology (Santa Cruz, CA).

#### Cell proliferation assay

Cells were plated in 12-well plates (3 × 10^4^ per well) and allowed to attach for 24 h. Cells were then treated with solvent control (DMSO) or varying concentrations of compounds and then trypsinized and counted after 24, 48 and 72 h using a Coulter Z1 particle counter.

#### Western blot analysis

Cells were plated in 6-well plates (3 × 10^5^ per well), allowed to attach for 24 h, and then treated with DMSO or varying concentrations of indicated compounds. Cells were lysed after 24 and 48 h and whole cell lysates were resolved on 7.5 or 12 % SDS-PAGE gels and proteins were transferred onto polyvinylidene difluoride membranes by wet electroblotting. Membranes were probed for indicated proteins by antibodies and β-actin was used as a loading control.

#### Flow cytometry

Cells were treated with 100 μM sulindac sulfide for indicated time and the general oxidative stress indicator CM-H_2_DCFDA (Invitrogen/Life Technologies, Waltham, MA) was used according to the manufacturer’s protocol. Fluorescence was measured by BD Accuri C6 flow cytometer (BD Biosciences, San Jose, CA) and data were analyzed according to the manufacturer’s guide.

#### Transfection and luciferase assay

Cells were plated in 12-well plates (1.5 × 10^5^ per well), allowed to attach for 24 h, and 400 ng of luciferase construct (pSp1, pSp3, pSurvivin, pVEGF or pMir27a-Luc) and 40 ng of β-galactosidase constructs (β-gal) with a constitutively active promoter were cotransfected into each well by Lipofectamine 2000 reagent (Invitrogen) and analyzed essentially as described [26].

#### Quantitative real-time PCR and TaqMan assay

Cells were treated as indicated and total RNA was extracted using RNeasy kit (Qiagen, Valencia, CA) then reverse transcribed using SuperScript reverse transcriptase (Invitrogen). Real-time PCR was carried out using a SYBR Green method (Applied Biosystems, Foster City, CA), and messenger RNA (mRNA) levels of target genes were normalized to that of the TATA-binding protein (TBP, as internal control). Total miRNA was extracted using mirVana isolation kit (Ambion/Life Technologies, Grand Island, NY) and TaqMan probes for miR-27a were purchased from the same company. TaqMan assay and analysis were carried out according to the manufacturer’s protocol.

#### Statistical analysis

Statistical significance of differences between experiment groups in cell proliferation, luciferase, flow cytometry, real-time PCR and TaqMan assays was analyzed using unpaired Student’s *t*-test and *P* value of <0.05 was considered statistically significant. Experiments were done in triplicate and all results are expressed as mean ± standard deviation (S.D.) for at least three independent determinations for each group.

## Results

**Fig. 1:**
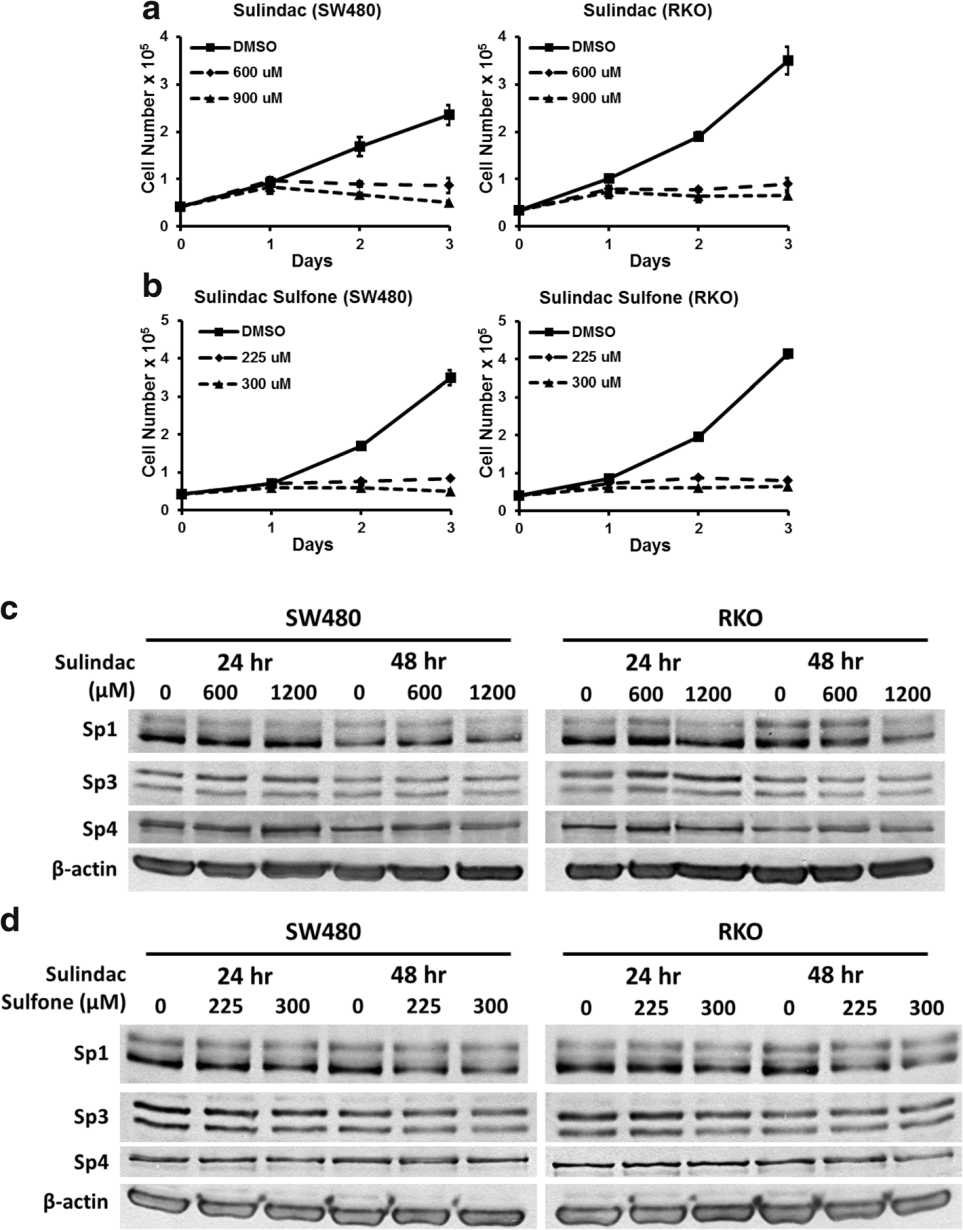
Sulindac and sulindac sulfone inhibit colon cancer cell growth without decreasing expression of Sp1, Sp3 and Sp4 proteins. **a**, **b** Sulindac and sulindac sulfone inhibit SW480 and RKO cell proliferation. Cells were treated with solvent control (DMSO), 600 or 900 μM sulindac, or 225 or 300 μM sulindac sulfone for 24, 48 and 72 h. Cell numbers were determined as described under Materials and Methods. Experiments were done in triplicate and results are expressed as mean ± S.D. for each determination. **c**, **d** Sulindac and sulindac sulfone have no effect on expression of Sp proteins in SW480 and RKO cells. Cells were treated with DMSO, 600 or 1200 μM sulindac, or 225 or 300 μM sulindac sulfone for 24 and 48 h. Sp1, Sp3 and Sp4 cellular levels were determined by Western blot analysis as described under Materials and Methods and β-actin was used as loading control

**Fig. 2:**
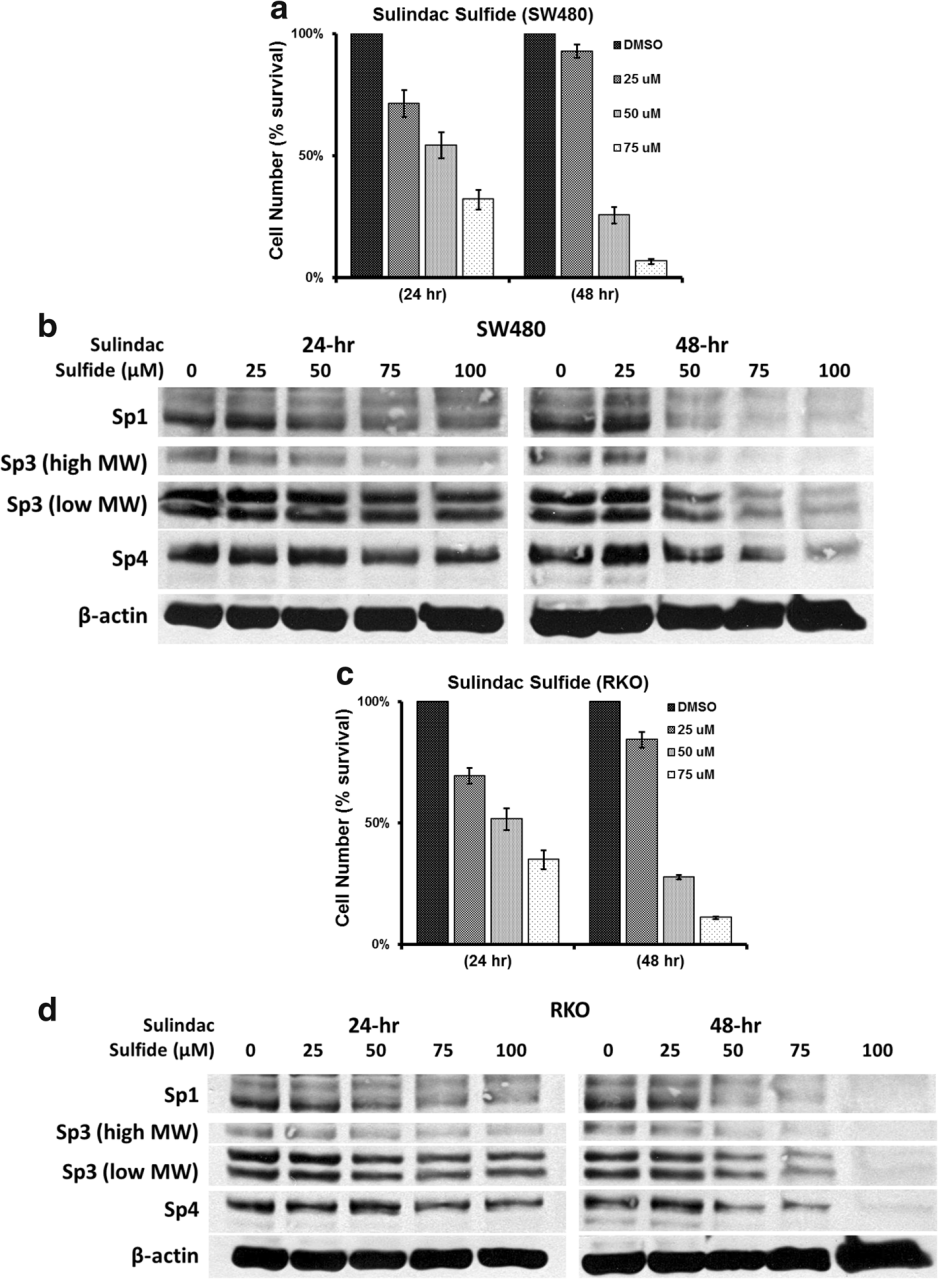
Sulindac sulfide inhibits colon cancer cell growth and decreases expression of Sp1, Sp3 and Sp4 proteins. **a**, **c** Sulindac sulfide inhibits SW480 and RKO cell proliferation. Cells were treated with DMSO, 25, 50, and 75 μM sulindac sulfide for 24 and 48 h. Cell numbers were determined as described under Materials and Methods. Experiments were done in triplicate and results are expressed as percentage of control (mean ± S.D.). **b**, **d** Sulindac sulfide decreases expression of Sp1, Sp3 and Sp4 proteins in SW480 and RKO cells. Cells were treated with DMSO, 25, 50, 75 and 100 μM sulindac sulfide for 24 and 48 h. Sp1, Sp3 and Sp4 cellular levels were determined by Western blot analysis as described under Materials and Methods and β-actin was used as loading control

Results illustrated in Fig. 1a and b show that sulindac and sulindac sulfone inhibited growth of SW480 and RKO cells at cytostatic concentrations between 600–900 and 225–300 μM, respectively. Western blot analysis of whole cell lysates from these cells indicated that 600 to 1200 μM concentrations of sulindac did not affect expression of Sp1, Sp3 and Sp4 proteins in SW480 and RKO cells after treatment for 24 and 48 h (Fig. 1c). Similar results were observed in these cells after treatment with 225 or 300 μM sulindac sulfone for 24 and 48 h (Fig. 1d) suggesting that growth inhibitory effects of these compounds was Sp-independent. Treatment of SW480 and RKO cells with 50 or 75 μM sulindac sulfide for 24 h inhibited cell proliferation (Fig. 2a and b) and also slightly decreased expression of Sp1, Sp3 and Sp4 proteins in SW480 and RKO cells (Fig. 2c and d). Sulindac sulfide induced similar responses after treatment for 48 h; however, at this time point, there was a pronounced downregulation of Sp1, Sp3 and Sp4 proteins in SW480 (Fig. 2c) and RKO (Fig. 2d) cells. Thus, sulindac sulfide was the most active sulindac derivative as observed in previous studies [33, 34] and the results suggest that the growth inhibitory effects of sulindac sulfide are correlated with downregulation of pro-oncogenic Sp proteins, and previous studies show that knockdown of one or more [35, 36] Sp proteins in colon cancer cells decreases cell cycle progression and induces apoptosis.

**Fig. 3:**
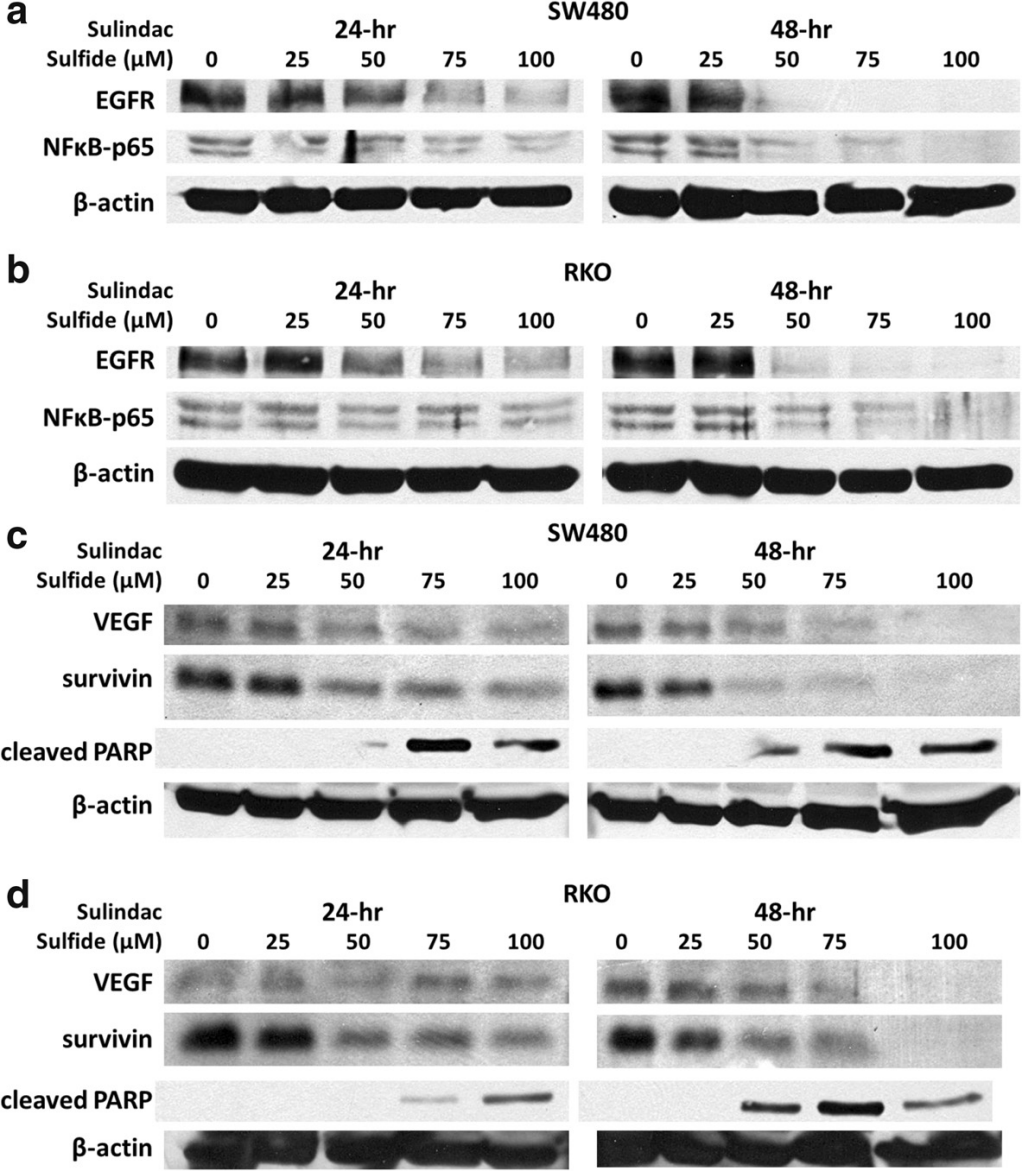
Sulindac sulfide decreases expression of EGFR, p65, VEGF and survivin and induces PARP cleavage in colon cancer cells. **a**, **b** Sulindac sulfide decreases expression of EGFR and the p65 subunit of NFκB in SW480 and RKO cells. Cells were treated with DMSO, 25, 50, 75 and 100 μM sulindac sulfide for 24 and 48 h. EGFR and p65 cellular levels were determined by Western blot analysis as described under Materials and Methods and β-actin was used as loading control. **c**, **d** Sulindac sulfide decreases expression of VEGF and survivin and induces PARP cleavage in SW480 and RKO cells. Cells were treated with DMSO, 25, 50, 75 and 100 μM sulindac sulfide for 24 and 48 h. VEGF, survivin and cleaved PARP cellular levels were determined by Western blot analysis as described under Materials and Methods and β-actin was used as loading control

We also investigated the effects of sulindac sulfide on Sp-dependent pro-apoptotic, growth inhibitory and anti-angiogenic responses in colon cancer cells. Results illustrated in Figs. 3a and b show that sulindac sulfide decreased EGFR expression in SW480 and RKO cells after treatment for 24 and 48 h and this is consistent with a decrease of EGFR mRNA (qPCR data not shown). We also examined the effects of sulindac sulfide on the p65 subunit of NFκB which is an Sp-dependent gene product in some cancer cell lines [26, 35, 37] and sulindac sulfide also decreased p65 expression in SW480 and RKO cells (Figs. 3a and b). In addition, sulindac sulfide also decreased expression of NFκB subunit p105 and upregulated expression of the NFκB inhibitor IκBα in SW480 and RKO cells (qPCR data not shown). Thus, sulindac sulfide-induced inhibition of SW480 and RKO cell proliferation was accompanied by downregulation of Sp1, Sp3, Sp4 and the Sp-dependent gene products, EGFR and p65. Treatment of SW480 cells with sulindac sulfide also decreased survivin expression and this was accompanied by caspase-dependent PARP cleavage which was observed after treatment for 24 and 48 h (Fig. 3c). Similar results were observed in RKO cells (Fig. 3d) and western blot data are in agreement with decrease of survivin transcript observed by qPCR (data not shown). It should be pointed out that the pro-apoptotic concentrations of sulindac sulfide were 25–50 μM in both cell lines with effective concentrations decreasing with increasing treatment times which is similar to that observed for sulindac sulfide-dependent downregulation of Sp1, Sp3 and Sp4. In addition, sulindac sulfide also decreased expression of the Sp-dependent angiogenic VEGF gene product in SW480 (Fig. 3c) and RKO (Fig. 3d) cells, demonstrating that sulindac sulfide-dependent downregulation of Sp1, Sp3 and Sp4 is accompanied by decreased expression of Sp-dependent growth promoting (EGFR), inflammatory (p65), survival (survivin) and angiogenic (VEGF) gene products. We also observed that knockdown of Sp1 by RNA interference inhibited RKO and SW480 cell growth by >60 % (data not shown) and this was consistent with previous reports showing that knockdown of Sp1 in these same cell lines resulted in a G0/G1 block in the cell cycle progression [36].

**Fig. 4:**
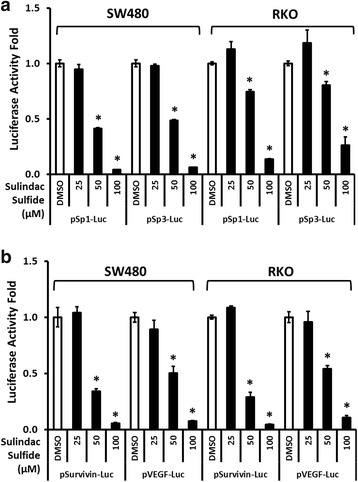
Sulindac sulfide decreases promoter gene activity of Sp1, Sp3, survivin and VEGF in colon cancer cells. **a** Cells were transfected with promoter-luciferase reporter constructs containing −751 bp promoter sequence of *SP1* gene (pSp1-Luc) or −417 bp of *SP3* gene (pSp3-Luc). **b** Cells were transfected with constructs containing −269 bp of *BIRC5* gene (pSurvivin-Luc) or −2018 bp of *VEGFA* gene (pVEGF-Luc). After 6 h of transfection, cells were treated with DMSO, 25, 50 and 100 μM sulindac sulfide for 13 h. Luciferase activity was determined as described under Materials and Methods. Experiments were done in triplicate and results are expressed as fold of control (mean ± S.D.). Asterisk (*) indicates statistical difference between control (DMSO) and treatment (*P* < 0.05)

We also examined the effects of sulindac sulfide on luciferase activity in cells transfected with reporter constructs containing GC-rich sequences from the Sp1 (pSp1-Luc) and Sp3 (pSp3-Luc) gene promoters [28, 29]. Sulindac sulfide decreased luciferase activity in both cell lines (Fig. 4a). Similar results were observed in cells transfected with the GC-rich survivin (pSurvivin-Luc) and VEGF (pVEGF-Luc) promoter-luciferase constructs [30, 31] (Fig. 4b), demonstrating that sulindac sulfide also decreased expression of Sp-regulated genes with GC-rich promoters. These results suggest a mechanism that involves transcriptional repression of GC-box-driven genes through sulindac sulfide-induced Sp downregulation.

**Fig. 5:**
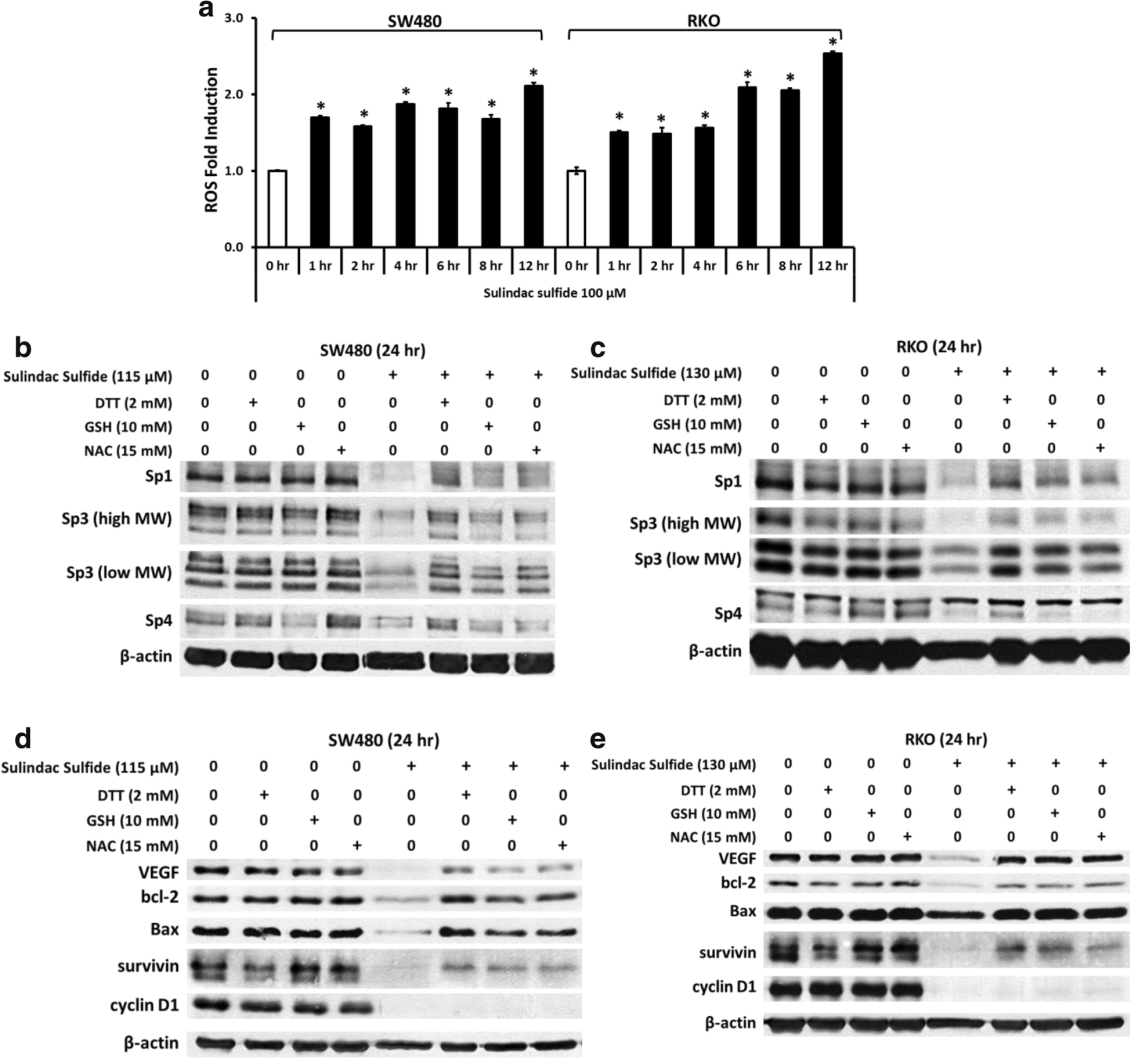
Sulindac induces ROS in colon cancer cells and reversal of sulindac sulfide-induced gene downregulation by thiol antioxidants. Sulindac sulfide increases cellular ROS level over time in SW480 and RKO cells. Sulindac sulfide decreases expression of Sp1, Sp3, Sp4, VEGF, bcl-2 and survivin and these effects are completely or partially reversed by antioxidants DTT, GSH and NAC cotreatment. Sulindac sulfide decreases expression of Bax in SW480 cells (reversible by antioxidants) but not in RKO cells. Sulindac sulfide also decreases expression of cyclin D1 and this is not reversible by antioxidants. **a** Cells were treated with 100 μM sulindac sulfide and cellular ROS level was measured by flow cytometry as described under Materials and Methods. Experiments were done in triplicate and results are expressed as fold of control (mean ± S.D.). Asterisk (*) indicates statistical difference between control (DMSO) and treatment (*P* < 0.05). **b**, **c** Cells were treated with DMSO, sulindac sulfide alone or in combination with antioxidants as indicated for 24 h. Antioxidants were pre-applied to cells for 45 min when used in combination with sulindac sulfide. Sp1, Sp3 and Sp4 cellular levels were determined by Western blot analysis. **d**, **e** VEGF, bcl-2, Bax, survivin and cyclin D1 were determined by Western blot analysis as described under Materials and Methods and β-actin was used as loading control

**Fig. 6:**
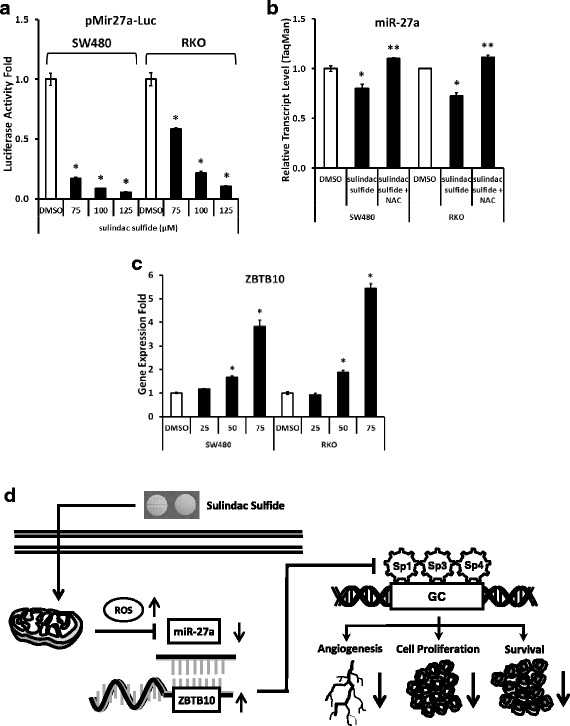
Effects of sulindac sulfide on miR-27a and ZBTB10 in colon cancer cells. **a** Sulindac sulfide decreases miR-27a promoter activity in SW480 and RKO cells. Cells were transfected with luciferase construct containing −638 bp promoter sequence of *miR-27a* gene (pMir27a-Luc). After 6 h, cells were treated with DMSO, 75, 100 and 125 μM sulindac sulfide for 18 h and luciferase activity was determined as described under Materials and Methods. **b** Sulindac sulfide decreases miR-27a transcript level in SW480 and RKO cells and this effect is reversed by antioxidant NAC. Cells were treated with DMSO, 100 μM sulindac sulfide alone or in combination with 10 mM NAC for 24 h and cellular level of the miR-27a transcript was determined by TaqMan PCR analysis as described under Materials and Methods. **c** Sulindac sulfide increases gene expression of ZBTB10 in SW480 and RKO cells. Cells were treated with DMSO, 25, 50 and 75 μM sulindac sulfide for 24 h and ZBTB10 mRNA levels were determined by real-time PCR analysis as described under Materials and Methods. All experiments were done in triplicate and results are expressed as fold of control (mean ± S.D.). Asterisk (*) indicates statistical difference between control (DMSO) and treatment, and double-asterisk (**) indicates statistical difference between single treatment and combination treatment (*P* < 0.05). **d** Effects of sulindac sulfide on the ROS-miR-27a-ZBTB10-Sp axis. Sulindac sulfide induces ROS; downregulates miR-27a; upregulates ZBTB10; downregulates Sp proteins and Sp-dependent survival/proliferative, inflammatory and angiogenic protein products; and induces growth inhibition and apoptosis

Several compounds including betulinic acid, tolfenamic acid and curcumin induce proteasome-dependent degradation of Sp proteins in prostate, pancreatic and bladder cancer cells respectively [22, 23, 38, 39]; however, proteasome inhibitors did not block downregulation of Sp1, Sp3 or Sp4 by sulindac sulfide in SW480 and RKO cells (data not shown). Recent studies have identified a role for ROS in mediating repression of Sp proteins and the nitro-NSAID GT-094 and curcumin induce ROS-dependent downregulation of Sp proteins and Sp-regulated gene products in colon cancer cells [25, 40]. Results illustrated in Fig. 5a show by flow cytometry that sulindac sulfide induced a time-dependent increase in ROS in SW480 and RKO cells. Figure 5b shows that sulindac sulfide downregulated Sp1, Sp3 and Sp4 proteins and this was partially reversed in SW480 cells cotreated with sulindac sulfide plus the antioxidant dithiothreitol (DTT). The other thiol antioxidants glutathione (GSH) and N-acetylcysteine (NAC) were less active than DTT as inhibitors of Sp downregulation in SW480 cells. However, in parallel experiments in RKO cells (Fig. 5c), DTT, GSH and NAC exhibited comparable activity as inhibitors of sulindac sulfide-induced downregulation of Sp1, Sp3 and Sp4 and the thiol antioxidants also inhibited the effects of sulindac sulfide on VEGF, bcl-2 and survivin proteins in SW480 (Fig. 5d) and RKO (Fig. 5e) cells. In contrast, expression of pro-apoptotic protein Bax was decreased by sulindac sulfide (and reversed by antioxidants) in SW480 cells (Fig. 5d) but not in RKO cells (Fig. 5e). These results indicate that the effect of sulindac sulfide on Bax is cell context-dependent. In contrast, thiol antioxidants did not block the effects of sulindac sulfide on cyclin D1, suggesting cyclin D1 is regulated in an ROS/Sp-independent manner in SW480 and RKO cells. Previous studies with ROS inducers suggest that the mechanism of Sp downregulation is associated with ROS-dependent repression of microRNA-27a (miR-27a) which results in the induction of the Sp repressor ZBTB10 [25, 40–42]. Treatment of SW480 and RKO cells with sulindac sulfide decreased both miR-27a promoter activity (Fig. 6a) and cellular level of miR-27a and this response was attenuated after cotreatment with antioxidant NAC (Fig. 6b). In a parallel experiment we observed that sulindac sulfide induced miR-27a-targeted ZBTB10 gene expression in SW480 and RKO cells (Fig. 6c). These results are consistent with previous studies in colon cancer cells showing that the miR-27a antagomir and ZBTB10 overexpression decrease expression of Sp1, Sp3 and Sp4 in RKO and SW480 cells [36]. Thus, like other ROS-inducing anticancer agents [25, 40–42], sulindac sulfide disrupts the miR-27a:ZBTB10 axis and the transcriptional repressor ZBTB10 competitively binds GC-rich *cis*-element to decrease expression of Sp1, Sp3, Sp4 and Sp-regulated genes (Fig. 6d).

## Discussion

Sp transcription factors Sp1, Sp3 and Sp4 are highly expressed in cancer cells/tumors and Sp1 is a negative prognostic factor for survival of gastric and pancreatic cancer and glioma patients [43–45]. Although Sp1 and other Sp proteins are important for early embryonic and postnatal development in mice, their expression is relatively low in adult tissues and there is evidence that Sp1 expression decreases with age in rodents and humans [46–48]. The functional importance of Sp1, Sp3 and Sp4 in cancer cells has been confirmed by RNA interference (RNAi) showing that knockdown of Sp transcription factors Sp1, Sp3 and Sp4 (singly or combined) decreases cell proliferation, survival, angiogenesis and inflammation [26, 35, 36, 42]. These results are consistent with identification (by RNAi) of several pro-oncogenic Sp-regulated genes important for cell growth (cyclin D1, EGFR, c-Met), survival (bcl-2, survivin), angiogenesis (VEGF and VEGF receptors), and inflammation (p65 subunit of NFκB) [22, 23, 26, 41, 42]. Thus, Sp transcription factors clearly contribute to the transformed cell phenotype and represent an example of non-oncogene addiction by cancer cells. Studies in this laboratory show that several structurally-diverse anticancer drugs downregulate Sp transcription factors through two major pathways, namely, degradation (activation of proteasomes or caspases) or by ROS-dependent transcriptional repression (Fig. 6d) and activation of one or both pathways is dependent on the agent and cell context. For example, previous studies with NSAIDs show that tolfenamic acid induced proteasome-dependent degradation of Sp proteins in pancreatic cancer [38], the nitro-NSAID GT-094 induced ROS-dependent repression [25], and aspirin induced caspase-dependent degradation of Sp1, Sp3 and Sp4 in colon cancer cells and in a mouse xenograft [35].

In this study, we also observed that sulindac and its metabolites inhibited proliferation of SW480 and RKO colon cancer cells and sulindac sulfide was the most active compound (Figs. 1a, b, 2a and c) and this was consistent with their relatively potent growth-inhibitory effects observed in other studies [29, 30]. At concentrations of sulindac or sulindac sulfone that inhibited SW480 and RKO cell proliferation, the levels of Sp1, Sp3 or Sp4 proteins were unchanged (Fig. 1c and d), whereas sulindac sulfide-dependent growth inhibition was accompanied by decreased expression of Sp1, Sp3 and Sp4 (Fig. 2b and d). These results clearly distinguish between sulindac sulfide and sulindac/sulindac sulfone and indicate that the anticancer activity of sulindac sulfide may be due, in part, to downregulation of Sp transcription factors.

Like tolfenamic acid and other compounds that induce Sp downregulation, sulindac sulfide also decreased expression of EGFR, survivin, VEGF and bcl-2 and also decreased the p65 subunit of NFκB which is Sp-regulated in only some cancer cell lines [26, 35, 37], and a previous study showed that high doses of sulindac also decreased survivin expression [49]. Tolfenamic acid induces proteasome-dependent downregulation of Sp1, Sp3 and Sp4 in pancreatic cancer cells [38]. However, proteasome inhibitors did not block sulindac sulfide-mediated repression of these transcription factors.

Studies with several ROS-inducing anticancer agents show that ROS-dependent transcriptional repression of Sp1, Sp3 and Sp4 (and Sp-regulated genes) is due to downregulation of miR-27a and/or miR-20a/17-5p [25, 40–42]. Decreased expression of these microRNAs results in induction of miR-targeted ZBTB10 and/or ZBTB4, which have been characterized as “Sp-repressors” that competitively displace Sp transcription factors from GC-rich promoter sites to decrease gene expression (Fig. 6d). In addition, similar results were observed in two cell lines (RKO and SW480) that have distinct genetic backgrounds, and previous studies in these cells with other agents that target Sp transcription factors also resulted in downregulation of Sp proteins [25, 35, 36, 40]. In addition, pharmacologic doses of ascorbate (which induces H_2_O_2_), *t*-butylhydroperoxide and H_2_O_2_ also decreased expression of Sp1, Sp3, Sp4 and Sp-regulated gene products in cancer cell lines [26, 50], and a number of other ROS-inducing agents including piperlongumine phenethylisothiocyanate and benzylisothiocyanate also downregulate Sp proteins [42].

In this study, sulindac sulfide was observed to induce ROS in SW480 and RKO cells in a time-dependent manner in flow cytometry experiments using an ROS indicator (Fig. 5). Results of our studies with sulindac sulfide are consistent with the ROS-dependent gene repression pathway where induction of ROS disrupts miR-27a-ZBTB10 interaction to decrease miR-27a and induce ZBTB10 (Fig. 6). ZBTB10 overexpression or miR-27a antagonism also decreases Sp protein expression [36, 51] and this is also consistent with sulindac sulfide-induced downregulation of Sp1, Sp3, Sp4 and Sp-regulated genes (Figs. 2 and 3).

## Conclusions

This study demonstrates that sulindac sulfide, the active metabolite of sulindac which is known to exhibit anti-neoplastic activity in human and experimental models of colon cancer, also downregulates Sp1, Sp3, Sp4 and pro-oncogenic Sp-regulated genes in colon cancer cells. The effects of sulindac sulfide also involve induction of ROS and the subsequent downregulation of miR-27a and induction of the "Sp repressor" ZBTB10, and this pathway has also been observed for other ROS inducers (Fig. 6d). It has previously been reported that high Sp1/Sp3 binding to the urokinase receptor predicted poor survival of colon cancer patients [52] and high expression of Sp1 was associated with an increased depth of invasion in another set of colon cancer patients [53]. A previous study showed that sulindac sulfide induced apoptosis in HUVEC cells [54]; however, a higher concentration (160 μM) was required, suggesting some specificity for cancer cell lines. Thus, the advantage/effectiveness of sulindac in colon cancer therapy may be due, in part, to the targeting of Sp transcription factors and it is possible that other anticancer agents or drug combinations that act, in part, through targeting Sp transcription factors may also be effective for colon cancer therapy.

## Abbreviations

COX: Cyclooxygenase
DMEM: Dulbecco’s modified Eagle’s medium
DMSO: Dimethyl sulfoxide
DTT: Dithiothreitol
EGFR: Epidermal growth factor receptor
GSH: Glutathione
miR-27a: microRNA-27a
NAC: *N*-acetylcysteine
NSAIDs: Non-steroidal anti-inflammatory drugs
RNAi: RNA interference
ROS: Reactive oxygen species
Sp: Specificity protein
VEGF: Vascular endothelial growth factor
